# The effect of human albumin administration on postoperative renal function following major surgery: a systematic review and meta-analysis

**DOI:** 10.1038/s41598-024-62495-0

**Published:** 2024-07-18

**Authors:** Kuen Su Lee, Ji Eun Kim, Giung Kang, Young Ju Won, Yoon Ji Choi

**Affiliations:** 1 Department of Anesthesiology and Pain Medicine, Eulji University Uijeongbu Eulji Medical Center, Eulji Uni-Versity School of Medicine, Uijeongbu, 11759 Republic of Korea; 2 Department of Anesthesiology and Pain Medicine, Ajou University School of Medicine, 164, World Cup-Ro, Yeongtong-Gu, Suwon, 16499 Republic of Korea; 3 Department of Anesthesiology and Pain Medicine, Korea University Guro Hospital, Korea University College of Medicine, Seoul, Republic of Korea; 4 Department of Anesthesiology and Pain Medicine, Korea University Ansan Hospital, Korea University College of Medicine, Seoul, Republic of Korea

**Keywords:** Acute kidney injury, Disease prevention

## Abstract

Optimal fluid management during major surgery is of considerable concern to anesthesiologists. Although crystalloids are the first choice for fluid management, the administration of large volumes of crystalloids is associated with poor postoperative outcomes. Albumin can be used for fluid management and may protect renal function. However, data regarding the effects of albumin administration on kidney function are conflicting. As such, the present study aimed to investigate the effect of albumin administration on renal function in patients undergoing major surgery and compare its effects with those of crystalloid fluid. The Embase, Medline, Web of Science, Cochrane Library, and KoreaMed databases were searched for relevant studies. The primary endpoint of the meta-analysis was the incidence of postoperative kidney injury, including acute kidney injury and renal replacement therapy. Twelve studies comprising 2311 patients were included; the primary endpoint was analyzed in four studies comprising 1749 patients. Perioperative albumin levels in patients undergoing major surgery did not significantly influence kidney dysfunction (p = 0.98). Postoperative fluid balance was less positive in patients who underwent major surgery and received albumin than in those who received crystalloids. Owing to the limitations of this meta-analysis, it remains unclear whether albumin administration during major surgery is better than crystalloid fluid for improving postoperative renal function.

## Introduction

The amount and type of intravenous fluid administered during major surgery are associated with perioperative outcomes and may affect patient prognosis^1^. Crystalloids and colloids are commonly used for fluid management in major surgical procedures. Although crystalloids are preferred for intravascular fluid management, large-volume intravascular crystalloid administration is associated with poor postoperative outcomes including delayed gastrointestinal function, multiple organ failure, morbidity, and mortality^2,3^. Albumin is the principal plasma protein and plays a central role in maintaining plasma oncotic pressure^4^; as such, it is used for fluid management in patients undergoing major surgery. According to Lazzareschi et al., albumin was used intraoperatively in approximately 15% of major non-cardiac surgeries in the United States between January 2014 and June 2020^5^. Other functions of albumin include the binding and transport of molecular substances and ligands, redox reactions, regulation of acid–base balance, capillary permeability, vascular integrity, and participation in apoptosis and homeostasis^6^.

Albumin can increase oncotic pressure and, consequently, preserve intravascular volume and renal perfusion pressure better than crystalloid fluids^7^. Albumin has also been hypothesized to improve renal function by affecting renal blood flow autoregulation by decreasing oxidative stress, endotoxemia, and endothelial stabilization^8^. However, results of studies investigating albumin administration vary, and conflicting conclusions have been drawn. Some studies have shown that administering albumin during the perioperative period in patients undergoing major surgery may prevent kidney injury^9,10^, whereas others have reported either a detrimental effect of albumin on the kidney, or no association with kidney function^11,12^. Considering the lack of consensus data, the effects of albumin administration on the kidneys during major surgeries remain uncertain.

As such, we aimed to compare the effect of albumin with that of crystalloid fluid administration on renal function in patients undergoing major surgery. This meta-analysis included randomized controlled trials (RCTs) in which albumin was administered during the perioperative period to adult patients undergoing major surgery.

## Results

### Description of studies

**Figure 1:**
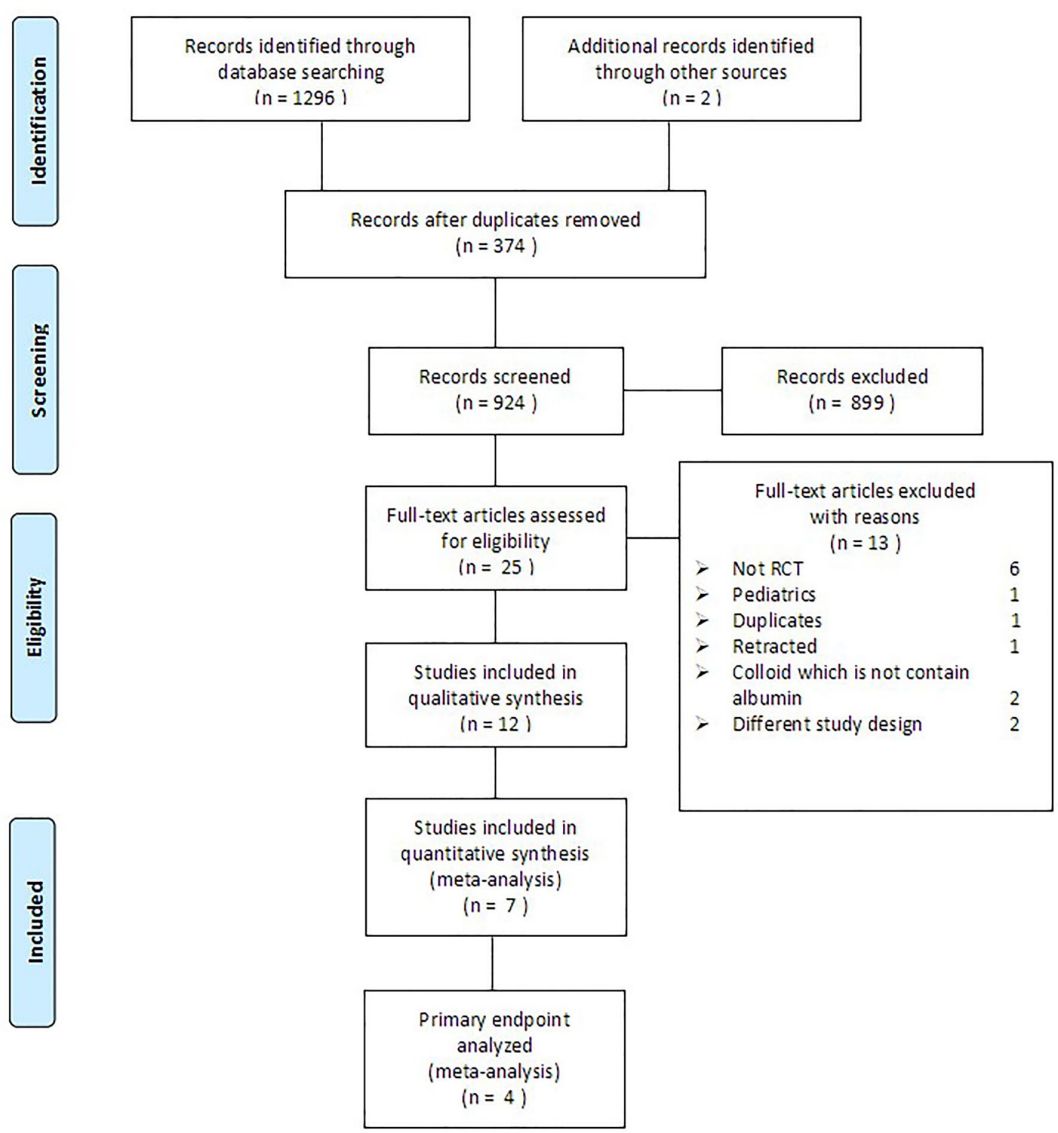
Flowchart of study selection for meta-analysis.

The initial literature search retrieved 1296 studies, and 2 were identified from other sources. A flow-diagram illustrating the study selection process is presented in Fig. 1. Of 25 potentially eligible studies, six were not RCTs, 1 was a pediatric study, 1 was a duplicate, 1 was retracted, 2 did not use albumin, and 2 had different study designs. Ultimately, therefore, 11 RCTs^13–24^ and 1 non-randomized control trial fulfilled the study inclusion criteria. Data regarding kidney dysfunction were obtained from the articles or via e-mail from the authors of these studies. The 6 studies for which kidney dysfunction data could not be collected^19–24^ included 278 patients, comprising 12.03% of the total number of patients across all studies fulfilling the study inclusion criteria. One study reported data regarding acute kidney injury (AKI) and renal replacement therapy (RRT)^14^.

### Study characteristics

Characteristics of the 12 included studies are summarized in Table 1.

**Table 1:** Characteristics of studies included in the meta-analysis.

Trial	Study type	Location	Population		Sample size	Intervention	Comparator	Timing
			**Inclusion criteria**	**Exclusion criteria**				
Abdallah et al. 2014 ^18^	RCT	Medical Sugar Center and Theodar Bilharz Research Institute, Cairo, Egypt	Patients with end-stage renal disease undergoing kidney transplantation	Patients with cardiac disease and liver dysfunction were excluded	44	Intravenous infusion of 20% human albumin with 0.9% normal saline	0.9% normal saline	Intraop
Bisgaard et al. 2020 ^13^	RCT	Aalborg University Hospital, Aalborg, Denmark	Adults aged 18 years or older undergoing elective upper gastrointestinal cancer surgery	Unsuitable for the use of the LiDCOplus system, contraindications for albumin, preoperative renal failure estimated glomerular filtration rate, pancreatic cancer and preoperative down staging using chemotherapy and/or radiation therapy, pregnancy	60	Received boluses of human albumin 5%	Ringer acetate	Intraop
Fiorica et al. 1991 ^19^	Nonrandomized control trial	University of South Florida College of Medicine, Tampa, Florida	Women underwent pelvic exenterations	Not stated	28	Received 100 g of a concentrated 25% albumin	D5.45% normal saline with 20 mEq of KCl/liter	Postop
Gallagher et al. 1985 ^20^	RCT	Deborah Heart and Lung Center, Browns Mills, New Jersey, and the Department of Anesthesia, University of Pennsylvania, Philadelphia, Pennsylvania	Patients scheduled for elective myocardial revascularization with aortocoronary saphenous vein grafts	Patients with significant left main coronary artery stenosis, poor left ventricular function (left ventricular end-diastolic pressure greater than 18 mm Hg or left ventricular ejection fraction less than 50% at catheterization, or both), or significant abnormalities in preoperative pulmonary function	10	5% albumin	Ringer lactate	Postop
Lee et al. 2016 ^14^	RCT	Asan Medical Center, University of Ulsan College of Medicine, Seoul, Korea	Patients scheduled for elective OPCAB were recruited for possible enrollment in the study, and those older than 20 years and of either sex had a serum albumin level of less than 4.0 g/dL the day before surgery	Preoperative renal dysfunction; a history of allergic reactions to HA; left ventricular ejection fraction of 40% or less; preoperative inotrope, intra-aortic balloon pump, or ventricular assist device support; or patients who required preoperative dialysis because of preexistent renal failure or who were undergoing repeat operations or concomitant valvular or aortic surgery	203	Administered 100, 200, or 300 mL of 20% HA at a rate of 5 mL/min according to the preoperative serum albumin level (3.5 to 3.9, 3.0 to 3.4, less than 3.0 g/dL, respectively)	0.9% NaCl	Intraop
Marelli et al. 1989 ^15^	RCT	The Montreal General Hospital/ McGill University, Montreal, Quebec, Canada	Adult patients undergoing intracardiac operations	Not stated	100	Received 200 mL of 25% albumin	Ringer lactate	Intraop
Pesonen et al. 2022 ^16^	RCT	Helsinki University Hospital, Helsinki, Finland	Aged 18 through 90 years undergoing the following primary or repeat open heart surgery procedure (elective surgery or surgery during the index admission) independently or in combination: coronary artery bypass graft surgery; aortic, mitral, or tricuspid valve replacement or repair; aortic root or ascending aorta surgery without hypothermic circulatory arrest; or the maze procedure	Immediate emergency surgery; congenital cardiac surgery; infection anticipated to compromise postprocedural rehabilitation; ongoing heart failure or low output syndrome (predefined significant inotropic support, mechanical ventilation, extracorporeal membrane oxygenation support, intra-aortic balloon pump, mechanical assistance of the left ventricle, left ventricular ejection fraction of less than 20%, or other comparable preoperative conditions); end-stage kidney disease (estimated glomerular filtration rate < 20 mL/min); hemophilia A or B; patient refusal of blood products or derivatives; and ticagrelor, prasugrel, clopidogrel, apixaban, or rivaroxaban use within 2 preoperative days or dabigatran use within 3 preoperative days	1386	4% albumin	Ringer acetate	Intraop
Rasmuassen et al. 2016 ^21^	RCT	Kirsten Cleemann Rasmussen, Rigshospitalet, Denmark	Patients > 18 years old, indication for elective post-renal operation including cystectomy, patients without anticoagulative, acetylsalicylic acid, or nonsteroidal anti-inflammatory drug treatment for the last 5 days	Intracerebral bleeding, manifest cardiac insufficiency, renal insufficiency demanding dialysis, hepatic or coagulation diseases	39	5% human albumin (25 mL/kg)	Ringer lactate	Intraop
Sade et al. 1985 ^22^	RCT	Medical University of South Carolina, Charleston, S. C	21 years of age or older, men and nonpregnant women. Each patient was scheduled to undergo an elective operation for coronary artery bypass grafting, valve replacement, or both	Pregnant or nursing	57	5% human serum albumin 800 mL/m^2^	Ringer lactate	Intraop
Scott et al. 1995 ^23^	RCT	St Vincent's Hospital, 41 Victoria Parade, Melbourne, Victoria 3064, Australia	Nonurgent patients presenting for first-time coronary artery bypass graft surgery with normal renal function (creatinine < 0.12 mmol/L) who were not on intravenous heparin or glyceryl trinitrate infusions and had not had aspirin within the preceding 10 days	Allergic to human albumin	64	Albumin 4.6% 1000 mL + plasmalyte 1000 mL	Plasmalyte	Intraop
Shah et al. 2014 ^24^	RCT	Institute of Kidney Diseases and Research Center, Civil Hospital Campus, Ahmedabad, Gujarat, India	Aged between 18 and 65 years, having an American Society of Anesthesiologist Physical Scoring risk between III or IV, scheduled for living donor renal transplantation	Age < 18 years, severe cardiovascular disease, liver dysfunction, and diabetes mellitus	80	100 mL of 20% human albumin	0.9% normal saline	Intraop
Skhirtladze et al. 2013 ^17^	RCT	Medical University of Vienna, Vienna, Austria	Elective cardiovascular surgery (i.e., coronary artery bypass grafting, valve repair or replacement, and surgery of the ascending aorta) on cardiopulmonary bypass	Disturbance in electrolytes	240	5% albumin up to 50 mL kg^-1^ day^-1^	Ringer lactate	Intraop

Five studies described the use of albumin at a concentration ≥ 20%^14,15,18,19,24^, whereas seven used albumin at a concentration ≤ 5%^13,16,17,20–23^. Seven studies included patients who underwent cardiac surgery^14–17,20,22,23^. Other surgery types included those for upper gastrointestinal cancer, pelvic exenteration, cystectomy, and renal transplantation.

Four studies did not use synthetic colloids^15,16,19,22^, and six did not indicate whether synthetic colloids were used^13,17,18,20,21,24^; for these, it was assumed that none were used. In one study, synthetic colloids were used intra-and postoperatively, but there was no difference in the volume of synthetic colloids used between the albumin and control groups^14^. One study used synthetic colloids during the postoperative period, and the amount of synthetic colloids administered in the albumin group was lower than that administered in the control group^23^.

In one study, albumin was used in the control group, albeit with a lower volume than that in the albumin group^22^.

### Risk of *bias* in the included studies

Two reviewers independently assessed the methodological quality of the studies using the Risk of Bias (ROB) tool recommended by the Cochrane Collaboration. Disagreements were resolved through discussion and consensus with another reviewer (YJ Choi).

**Figure 2:**
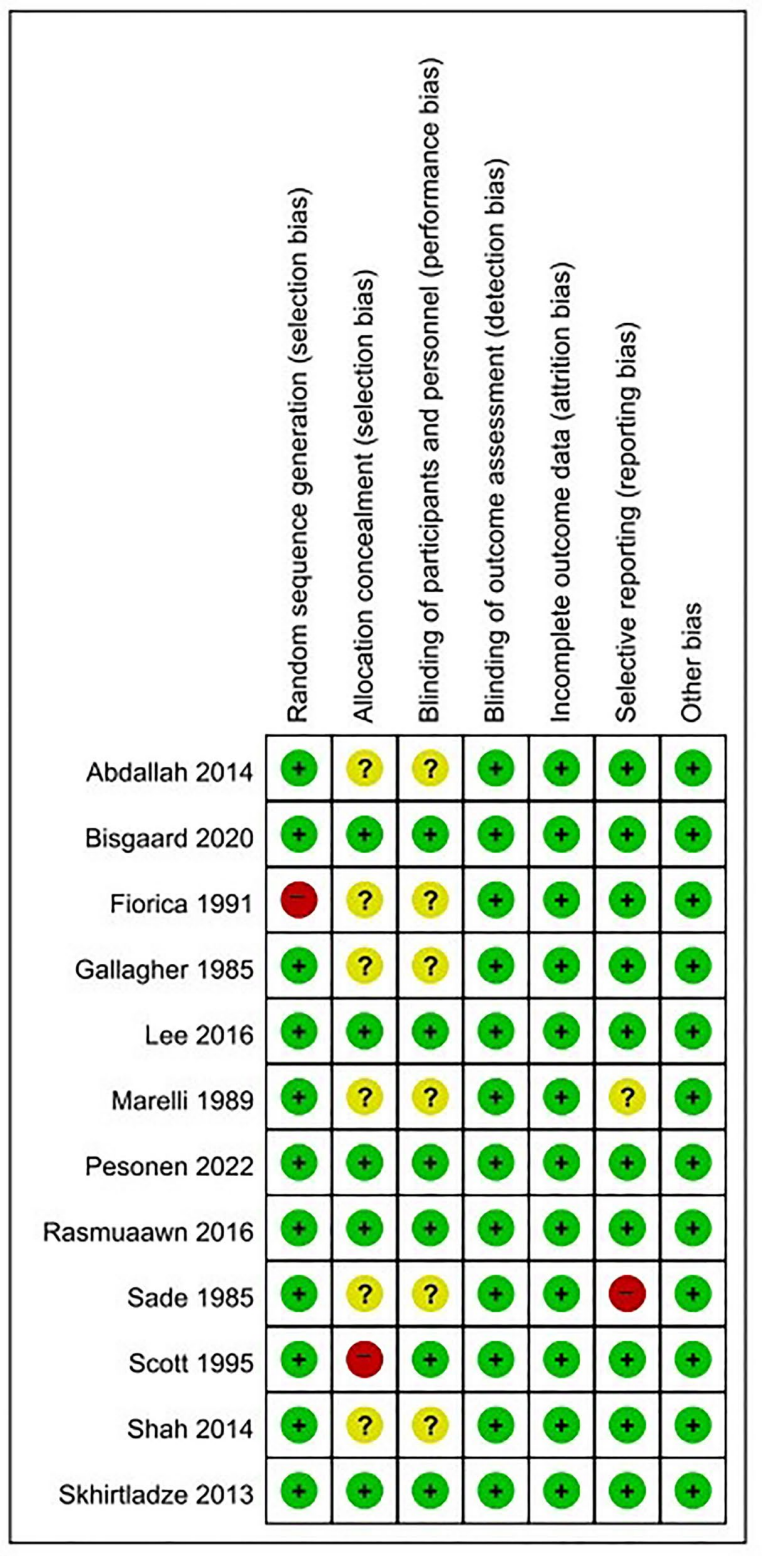
Risk of bias summary: The judgment of the review author on each risk of bias item is presented in different colors. Green, yellow, and red circles indicate low, some concerns, and high risks of bias, respectively.

The Cochrane tool was used to determine risk of bias, which was evaluated as “low”, “high” or “some concern”, as shown in Fig. 2.

### Kidney dysfunction

Six studies reported kidney dysfunction in patients in whom albumin was administered during major surgeries. Four of the six studies reported data regarding the incidence of AKI (n = 84)^13–16^. Bisgaard et al. defined AKI based on the Risk, Injury, and Failure; and Loss; and End-stage kidney disease (RIFLE) criteria. Lee et al. defined AKI in accordance with the Acute Kidney Injury Network (AKIN) criteria. The criteria for AKI were not clearly defined in the study by Marelli et al. A study by Pesonen et al. defined AKI as an increase in postoperative creatinine levels to at least twice the preoperative level. Four patients required RRT in three of six studies^14,17,18^, which was too small a sample for meaningful statistical analysis; therefore, RRT data were excluded from the comparison between the two groups. Four studies reported creatinine data^17,18,23,24^; however, changes creatinine levels over 48 h or 7 days for individual patients could not be obtained; therefore, creatinine data were excluded.

**Figure 3:**
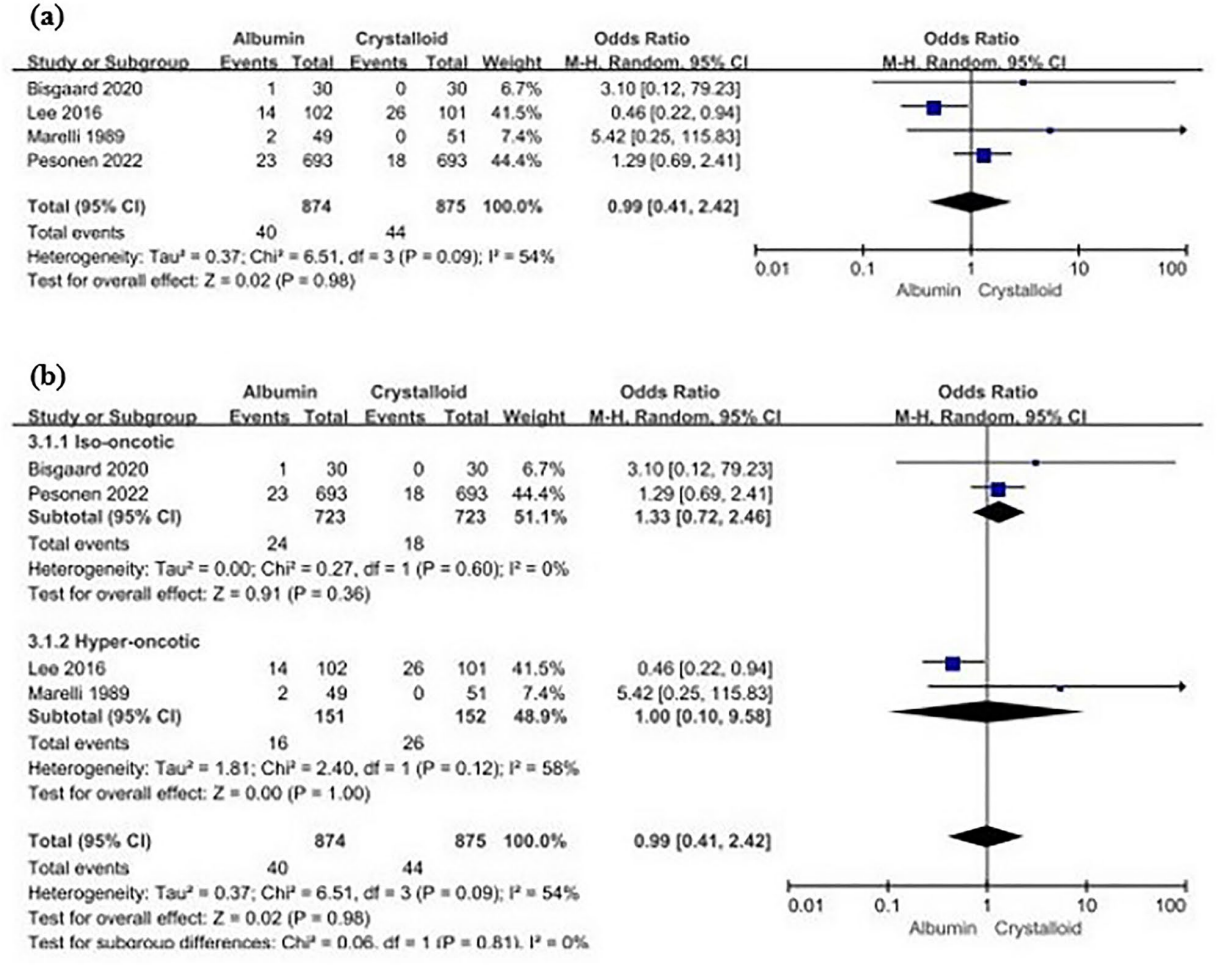
Forest plots of studies reporting kidney dysfunction in patients who underwent major surgery. (**a**) all studies, (**b**) albumin concentration. *CI* confidence interval, *M-H* Mantel − Haenszel test, *df* degrees of freedom.

Albumin administration did not significantly affect kidney function compared with crystalloid administration (albumin, 40/874 [4.58%] versus [vs.] crystalloid, 44/875 [5.03%]; odds ratio [OR] 0.99 [95% confidence interval (CI) 0.41 − 2.42]; I^2^ = 54%, p = 0.98) (Fig. 3A).

In the subgroup analysis according to different albumin concentrations (i.e., iso-oncotic and hyper-oncotic), studies using ≤ 5%^13,16^ and > 20%^14,15^ albumin were classified into the iso-oncotic and hyper-oncotic groups, respectively. Administration of albumin at both iso-oncotic and hyperoncotic concentrations did not significantly affect the incidence of kidney dysfunction when compared with the effect of crystalloid administration (iso-oncotic group, albumin, 24/723 [3.32%] vs. crystalloid, 18/723 [2.49%], OR 1.33 [95% CI 0.72 − 2.46], I^2^ = 0%, p = 0.36; hyperoncotic group, albumin, 16/151 [10.60%] vs. crystalloid, 26/152 [17.11%], OR 1.00 [95% CI 0.10 − 9.58], I^2^ = 58%, p = 1.00) (Fig. 3B).

### Mortality and intensive care unit stay

**Figure 4:**
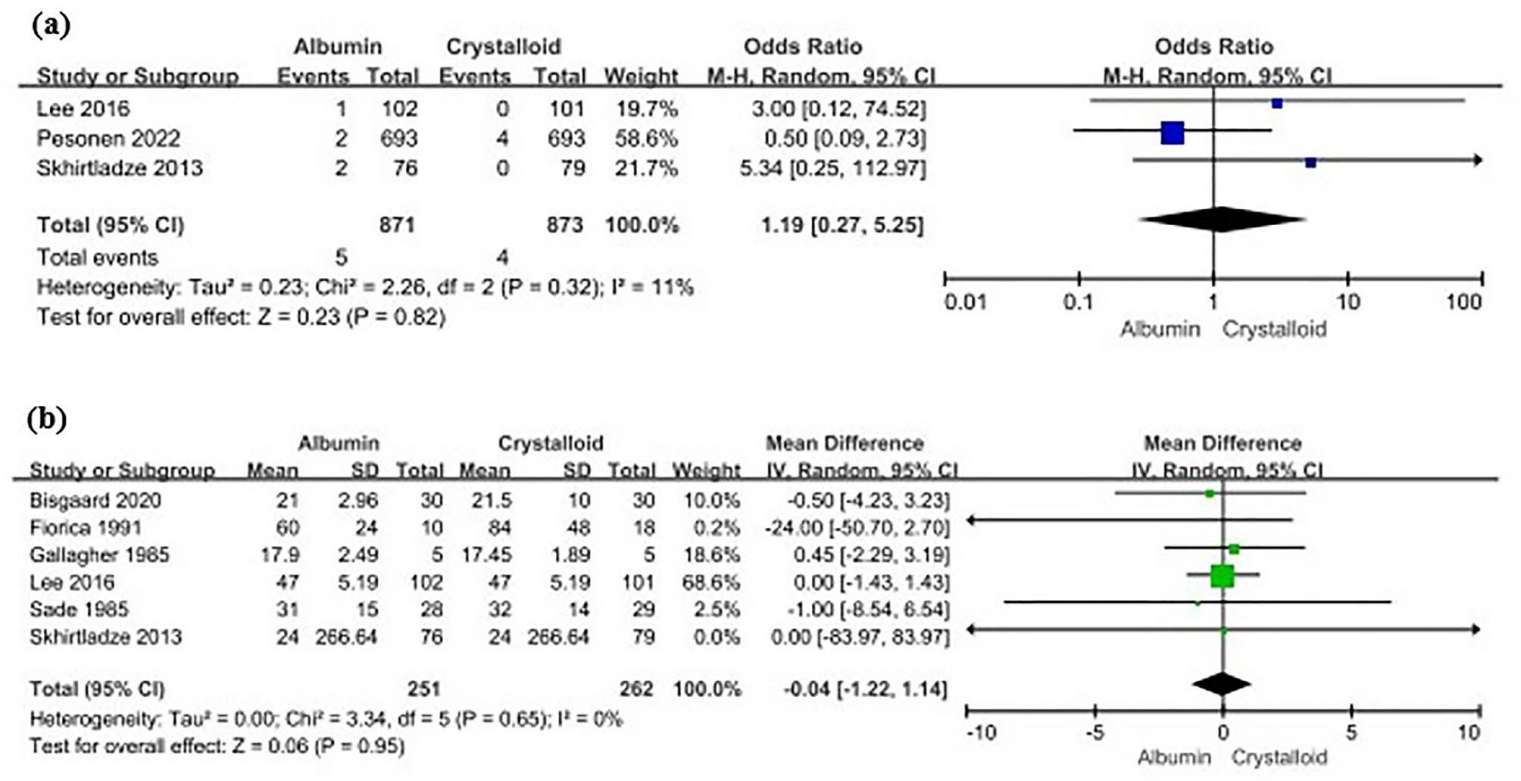
Forest plots showing (**a**) odds ratio of mortality and (**b**) mean difference of intensive care unit stay (h) in albumin vs. crystalloid use in patients who underwent major surgery. *CI* confidence interval, *M-H* Mantel − Haenszel test, *SD* standard deviation, *IV* inverse variance.

Three^14,16,17^ and six^13,14,17,19,20,22^ studies reported mortality and intensive care unit (ICU) stay, respectively, in patients who underwent major surgery and were administered albumin. Albumin administration did not significantly influence mortality (albumin, 5/871 [0.57%] vs. crystalloid, 4/873 [0.46%]; OR 1.19 [95% CI 0.27 − 5.25]; I^2^ = 11%, p = 0.82) (Fig. 4A). The pooled mean difference (MD) of the ICU stay was -0.04 (95% CI − 1.22 to 1.14; I^2^ = 0%, p = 0.95) between the albumin and crystalloid groups (Fig. 4B).

### Other outcomes

Secondary outcome data, including the studies, number of participants, risk ratio or MD, and p-values, are summarized in Table 2.

**Table 2:** Secondary outcome data from studies included in the meta-analysis.

Outcome	Studies included (n = 12)	Participants	Risk ratio or mean difference (95% CI)	I^2^ (%)	P-value	Quality of evidence (GRADE)	References
Intraoperative fluid balance	5	1650	− 517.63 (− 1112.07 to 76.80)	94	0.09	⊕ ⊕ ⊖ ⊖	^13,16,17,20,21^
Postoperative fluid balance	5	317	− 657.46 (− 1257.24 to − 57.68)	97	0.03	⊕ ⊕ ⊖ ⊖	^13,17,19,20,23^
Intraoperative blood loss	4	1585	29.35 (− 134.06 to 192.77)	66	0.72	⊕ ⊕ ⊖ ⊖	^13,15,16,21^
Postoperative blood loss	7	1935	− 16.06 (− 156.84 to 124.72)	97	0.82	⊕ ⊖ ⊖ ⊖	^13,14,16,17,20,22,23^
Intraoperative RBC transfusion	8	2001	0.37 (0.07 to 0.67)	80	0.01	⊕ ⊕ ⊖ ⊖	^13−17,20−22^
Postoperative RBC transfusion	6	549	− 0.45 (− 1.29 to 0.39)	90	0.29	⊕ ⊖ ⊖ ⊖	^13,14,17,20,22,23^
Lowest hemoglobin level	6	621	− 1.29 (− 2.12 to − 0.46)	95	0.002	⊕ ⊕ ⊖ ⊖	^13–15,17,21,23^
Postoperative pulmonary edema	5	487	0.70 (0.28 to 1.74)	0	0.45	⊕ ⊕ ⊕ ⊕	^13–15,18,24^

⊕  ⊕  ⊕  ⊕ , high quality of evidence; ⊕  ⊕  ⊕  ⊝ , moderate quality of evidence; ⊕  ⊕  ⊝  ⊝ , low quality of evidence, ⊕  ⊝  ⊝  ⊝ , very low-quality of evidence. GRADE, Grades of Recommendation, Assessment, Development, and Evaluation, RBC: red blood cell.

### Intraoperative and postoperative fluid balance

Among the included studies investigating patients who underwent major surgery and received albumin, five and five reported results on intraoperative and postoperative fluid balance, respectively. Albumin administration did not significantly influence intraoperative fluid balance (MD − 517.63 [95% CI − 1112.07 to 76.80]; I^2^ = 94%, p = 0.09). Compared with crystalloids, albumin administration significantly lowered postoperative fluid balance (MD − 657.46 [95% CI − 1257.24 to − 57.68]; I^2^ = 97%, p = 0.03).

### Intraoperative and postoperative blood loss

Four and seven studies reported intra- and postoperative blood loss, respectively, in patients who underwent major surgery and albumin therapy. Compared with crystalloid, albumin administration did not significantly affect intraoperative (MD 29.35 [95% CI − 134.06 to 192.77]; I^2^ = 66%, p = 0.72) and postoperative (MD − 16.06 [95% CI − 156.84 to 124.72]; I^2^ = 97%, p = 0.82) blood loss.

### Intraoperative and postoperative red blood cell transfusion

Eight and six studies reported on intraoperative and postoperative red blood cell (RBC) transfusions, respectively, in patients who underwent major surgery and were administered albumin. Intraoperative RBC transfusion was significantly higher in patients who received albumin than in those who received crystalloids (MD 0.37 [95% CI 0.07 to 0.67]; I^2^ = 80%, p = 0.01). In contrast, the volume of postoperative RBC transfusions did not significantly differ between patients who received albumin versus those who received crystalloids (MD -0.45 [95% CI − 1.29 to 0.39]; I^2^ = 90%, p = 0.29).

### Lowest hemoglobin level

Six studies reported nadir perioperative hemoglobin levels. Compared with the crystalloid group, the nadir hemoglobin level was significantly lower in the albumin group (MD − 1.29 [95% CI − 2.12 to − 0.46]; I^2^ = 95%, p = 0.002).

### Postoperative pulmonary edema

Five studies reported on postoperative pulmonary edema. Albumin administration did not significantly influence postoperative pulmonary edema in the albumin and crystalloid groups (albumin, 9/243 [3.70%] vs. crystalloid, 13/244 [5.33%]; OR 0.70 [95% CI 0.28 − 1.74]; I^2^ = 0%, p = 0.45).

### Sensitivity analysis and publication *bias*

Sensitivity analysis was performed to evaluate the influence of synthetic colloids by excluding studies in which synthetic colloids were used or those in which their use was not described. As reported in Table 1, synthetic colloids did not influence kidney dysfunction. Based on Egger’s test performed to evaluate publication bias in this meta-analysis, the funnel plot displayed no obvious asymmetry (Fig. 2). Furthermore, Egger’s test indicated no evidence of publication bias in studies addressing the kidney dysfunction endpoint (t = 1.85, p = 0.138). The quality of evidence for kidney dysfunction, mortality, and ICU stay was low, high, and high, respectively. The overall quality of evidence for the other outcomes, assessed using the Grading of Recommendations, Assessment, Development, and Evaluations (GRADE) criteria, is presented in Table 2.

## Discussion

This meta-analysis synthesized evidence from RCTs comparing the effects of crystalloid versus albumin administration on renal function in patients who underwent major surgery, with results revealing no association. Several meta-analyses of mortality following the administration of crystalloids and albumin under various clinical conditions have been conducted^25,26^. Although individual studies have reported the effects of albumin administration on kidney function during major surgery, no meta-analysis has been performed and the effects of albumin administration on the kidneys during the perioperative period remain undefined.

The incidence of mortality in the broad population of patients undergoing major surgery is approximately 1–4%, suggesting that mortality is a relatively insensitive outcome^27^. In contrast, the incidence of morbidity is usually much higher than that of mortality, and morbidity can provide results that are more sensitive to meta-analyses than mortality as an outcome^28,29^. Moreover, postoperative kidney injury is a continual medical concern that significantly influences prognosis. Therefore, this study aimed to understand the effects of albumin administration on kidney function in patients undergoing major surgery.

In a retrospective observational study involving patients receiving albumin-containing fluids for fluid bolus therapy, the use of hyperosmolar albumin resulted in less volume, less sodium and chloride, and fewer adverse outcomes than the use of iso-oncotic albumin^30^. Previously, Wiedermann et al. reported that hyperoncotic albumin reduced the probability of AKI by 76% and suggested that albumin exhibits renoprotective properties^31^. In contrast, Schortgen et al. reported the possibility of harmful effects on renal function and poorer outcomes with hyperosmolar albumin^32^. However, results of the present study contradict those reported in these two studies, and may be explained by differences in patient condition(s) in the pooled studies. The present review included studies that focused on patients who underwent surgery. Wiedermann et al. included not only studies that focused on patients who underwent surgery, but also those that focused on patients with cirrhosis (one study on patients who underwent surgery and six on patients with cirrhosis). Albumin administration improves kidney function and prevents AKI in patients with cirrhosis^33^. Furthermore, in contrast to the present study, in which studies using a mixture of iso-oncotic and hyper-oncotic albumin were pooled, Wiedermann et al. used only hyper-oncotic albumin. To verify the results of the study by Wiedermann et al., it is necessary to carefully and cautiously analyze the effects of hyperoncotic albumin on kidney function. Schortgen et al. selected patients already in a state of multiple organ failure and severe hemodynamic instability. These patients may have advanced multiple organ failure and hemodynamic instability; therefore, their physical conditions were different from those of patients who underwent major surgery. Furthermore, there were differences at baseline, with the crystalloid group including significantly more medical patients and a lower volume of fluid than the albumin group. In a retrospective study by Kim et al., no significant association was observed between hyperoncotic albumin levels and AKI in patients who underwent major abdominal surgery^34^. In contrast, in a retrospective study by Udeh et al., hyperoncotic albumin in postoperative shock appeared to be associated with AKI^35^.

Although there was no significant difference in blood loss volume between patients administered albumin versus crystalloids during major surgery in our study, the volume of RBC transfusions was higher in the albumin group. This could, in part, be explained by lower perioperative hemoglobin levels in the albumin versus crystalloid group. Other studies reported no difference in blood loss between the albumin and crystalloid groups; however, hemoglobin level was lower in the albumin group^36,37^. Albumin has a greater and longer-lasting plasma volume expansion effect than crystalloid fluid^38^. In this meta-analysis, we speculated that, despite no significant difference in blood loss between patients administered albumin versus crystalloids during major surgery, those receiving albumin underwent a significantly higher volume of RBC transfusions than those receiving crystalloids due to the overestimated blood loss caused by hemodilution resulting from the administered albumin. A recent network meta-analysis reported that an albumin priming strategy resulted in lower postoperative hemoglobin counts than crystalloids, despite no significant difference in postoperative blood loss in on-pump cardiac surgery^39^. This study showed that albumin priming resulted in more perioperative RBC transfusions than crystalloid priming. Albumin is known to cause more hemodilution than crystalloids. In a study by Arya et al., the crystalloid group was infused with a volume three times that of the albumin group and the immediately measured hemoglobin level was lower in the crystalloid group than in the albumin group. However, over time, the measured hemoglobin levels were lower in the albumin group than in the crystalloid group^40^. Caution is necessary when interpreting these results because the cause of low hemoglobin levels elicited by albumin administration and RBC transfusion has not been established.

In our meta-analysis, there was no statistically significant difference in intraoperative fluid balance between the albumin and crystalloid groups among patients who underwent major surgery. However, our results should be interpreted with caution. This is because the study by Gallagher, which was the only of the five studies to report a greater amount of intraoperative fluid in the albumin group than in the crystalloid group, excluded a portion of blood loss when calculating fluid balance.

Another review reported that the albumin group exhibited a less positive fluid balance than the crystalloid group in critically ill patients^41,42^. Albumin can efficiently maintain intravenous fluids because its molecular weight is larger than that of crystalloid fluids^43^. Yanase et al. reported that fluid bolus therapy using albumin had a more sustained effect than crystalloids on mean arterial, central venous, and perfusion pressures in patients undergoing cardiac surgery^44^. It has been reported that the volume of fluid required to achieve the same resuscitation endpoint is lower in the albumin group than in the crystalloid group^45,46^. Nevertheless, there is still controversy as to whether albumin versus crystalloid is beneficial. During resuscitation for septic shock, the volume of fluid required when albumin and crystalloid fluids were administered in a blinded fashion was similar between the two groups^47,48^.

In our meta-analysis, the crystalloid group exhibited a significantly higher postoperative positive fluid balance than the albumin group. Xu et al. reported that perioperative fluid infusion was lower in the albumin group than crystalloid group^49^. Positive perioperative fluid balance adversely affects renal function and patient prognosis^50^. Firth et al. reported that fluid-extended animals exhibit increased renal venous pressure, resulting in elevated interstitial pressure and reduced renal blood flow compared with fluid-depleted animals^51^. In a meta-analysis, although there was a trend toward lower volumes of albumin administered compared with crystalloids in ICU patients, which was not statistically significant, the central venous pressure was significantly higher with albumin than with crystalloids^52^. Additionally, Nishimoto et al. reported that the higher the positive fluid balance, the higher the incidence of AKI^53^. Nishimoto et al. reported that an increase in the probability of AKI is associated with an increase in the intraoperative fluid balance > 40 ml/kg. However, some of the studies included in the present meta-analysis reported a mean intraoperative fluid balance < 40 ml/kg, assuming a mean adult weight of 60 kg. Furthermore, in the study by Nishimoto et al., the expected probabilities of postoperative AKI were 0.05 and 0.17 when the intraoperative fluid balance was 50 ml/kg and 100 ml/kg, respectively. However, among the studies included in the present meta-analysis, the study with the largest difference in mean intraoperative fluid balance between the albumin-administered and crystalloid groups was 21 ml/kg, assuming an average adult weight of 60 kg. This explains the observed (in the present meta-analysis) lack of any significant difference in kidney dysfunction and the presence of a significant difference in intraoperative fluid balance between the albumin and crystalloid groups.

The present meta-analysis had several limitations. The effect of albumin administration on kidney function based on albumin concentration is ambiguous because this meta-analysis included both iso-oncotic and hyper-oncotic albumin studies. However, in the subgroup analysis, the effects of iso-oncotic and hyper-oncotic albumin on kidney function were not different.

In addition, the effect of albumin may be influenced by additional conditions such as the timing of albumin administration or other clinical factors in patients who undergo major surgery. Zhang et al. reported that administration of albumin in the ICU after cardiac surgery decreased mortality^54^. The authors suggested that albumin administration may be beneficial for ICU patients with hypovolemia, an inflammatory state, capillary leakage, vasodilation, and a high degree of vascular permeability after cardiac surgery. Our study found no significant impact of administered albumin compared with crystalloids on mortality in patients undergoing major surgery. However, due to its low incidence, definitive conclusions regarding the effects of albumin on mortality cannot be drawn. Vincent et al.^55^ reported that albumin could potentially improve morbidity when used to treat hypovolemia secondary to trauma and surgery.

Moreover, there was a risk of bias in the population or type of surgery, and the low level of evidence was due to the inconsistent AKI criteria based on RIFLE and AKIN used in the included studies. In this meta-analysis, the primary endpoint was analyzed in only four studies involving approximately 1700 patients. One of the four studies included approximately 80% of the patients. Furthermore, the primary endpoint was analyzed in patients who underwent heterogeneous major surgeries, including cardiac and non-cardiac procedures. Epidemiologically, postoperative AKI after cardiac and non-cardiac surgery is a common cause of renal hypoperfusion, inflammation, oxidative stress, exogenous and endogenous toxins, ischemia, and reperfusion injury^56,57^. We investigated the incidence of AKI in a heterogeneous population undergoing major surgery and found that, except for cardiac surgery, other major surgeries had similar rates of AKI^58^. Therefore, in this meta-analysis, a search was performed to identify major surgeries, including cardiac and noncardiac procedures. Many clinical studies investigating postoperative AKI have been published, mainly using the KDIGO, RIFLE, and AKIN criteria; however, to date, there has been no clear gold standard for postoperative AKI^59^. Therefore, despite the lack of a consistent definition of AKI, we included all studies with AKI data. Owing to the risk of bias in the population or type of surgery, and the lack of a consistent definition of AKI in this meta-analysis, future high-quality randomized controlled trials are necessary to determine the efficacy of administered albumin on kidney function in patients undergoing major surgery.

In conclusion, this meta-analysis has important policy implications for fluid management during major surgical procedures. Results of this meta-analysis suggested that kidney function may not be affected by albumin, and no clear advantage of albumin over crystalloid fluid administration during major surgery was observed. However, owing to the limitations of this meta-analysis, a larger trial investigating the effects of albumin on kidney function in patients undergoing major surgery should be conducted. In addition, albumin is expensive compared to crystalloid fluids; hence, the medical and economic burden is high. Albumin should be used cautiously during major surgeries until more conclusive studies are completed.

## Method

Institutional review board approval was not required for this systematic review and meta-analysis. The systematic review was performed according to the Cochrane Review Methods and a previously registered protocol (PROSPERO, registration number CRD42021259805), and reported in accordance with the Preferred Reporting Items for Systematic Reviews and Meta-Analyses (i.e., “PRISMA) statement.

### Identification of relevant studies

Query searches identified relevant studies in the Embase, Medline, Web of Science, Cochrane Library, and KoreaMed databases that were published from inception up to December 31, 2022. The detailed search strategy is illustrated in Fig. 1.

A systematic search for relevant published trials was performed without language restrictions. In addition, the reference lists of relevant review articles were also searched for potentially eligible studies.

Studies fulfilling the following criteria were considered to be eligible: RCTs; a population of patients who underwent major surgery, defined as any cardiac, thoracic, major vascular, intra-abdominal, or retroperitoneal procedure; perioperative administration of intervention fluid (albumin: 4–5%, 20%, or 25% human albumin) vs. control (crystalloid fluid: 0.9% normal saline, Ringer’s acetate solution, PlasmaLyte, or Hartmann’s solution); and reported kidney-related clinical outcomes as primary or secondary outcomes.

Studies with patient populations < 18 years of age, and those including minor surgeries, defined as invasive procedures not requiring a large surgical incision that would be expected to result in significant bleeding^60^, were excluded.

### Types of outcome measures

A review of relevant articles identified AKI, RRT, and changes in serum creatinine levels as parameters of postoperative kidney injury. In cases in which both AKI and RRT data were described in the article, AKI data were selected as the primary outcome.

### Data collection and analysis

Full-text copies of all relevant studies were independently assessed based on predefined inclusion criteria. Two authors (KS Lee and YJ Choi) independently extracted the data and YJ Won verified accuracy. The extracted data included trial design features, perioperative laboratory values, intraoperative fluid volumes, complications (AKI, RRT, and pulmonary edema), mortality, and length of ICU stay. The methodological quality across statistically pooled outcomes was evaluated using the GRADE guidelines^61^.

### Risk of *bias* in included studies

The risk of bias was analyzed using the Cochrane Risk of Bias Tool (ROB v2.0), which has five domains: bias arising from the randomization process; bias due to deviations from intended interventions; bias due to missing outcome data; bias in the measurements of the outcome; and bias in the selection of the reported results. Each domain classifies studies into “low,” “some concern,” and “high” risk of bias. Two authors (KS Lee and YJ Won) independently evaluated the methodological quality and risk of bias. Any disagreements were discussed with another author (YJ Choi).

### Statistical analysis

Review Manager version 5.4 (Cochrane Collaboration, Oxford, United Kingdom) was used for data analysis and synthesis. Results expressed as median and interquartile range were calculated as mean and standard deviation using the methods described by Wan et al. ^62^. Depending on the reported effect size measures, pooled risk ratios, MD, and 95% CI were calculated. A random-effects approach (inverse variance or Mantel–Haenszel) was selected to allow for expected heterogeneity across studies because data collected from different study designs and surgeries would not satisfy the assumption of a fixed-effects meta-analysis. The degree of heterogeneity among the studies was based on I-squared statistics, with ranges of 0–50%, 50–75%, and 75–100% considered to be low, moderate, and high degrees of heterogeneity, respectively. A sensitivity analysis was performed by removing studies that used synthetic colloids or did not mention the use of synthetic colloids in the meta-analysis to determine whether synthetic colloids could alter the results. A funnel plot was used to evaluate potential publication bias, and Egger’s test was performed to evaluate funnel plot asymmetry using STATA Release 17 (StataCorp LLC, College Station, TX, USA).

### Supplementary Information

Supplementary Figure 1.

Supplementary Figure 2.

Supplementary Table 1.

## Data Availability

All data generated or analysed during this study are included in this published article.
