# Supplementary figures and images for: The effect of human albumin administration on postoperative renal function following major surgery: a systematic review and meta-analysis
Source: Sci Rep. 2024 Jul 18;14:16599. doi: 10.1038/s41598-024-62495-0 (PMC11258253; doi:10.1038/s41598-024-62495-0)

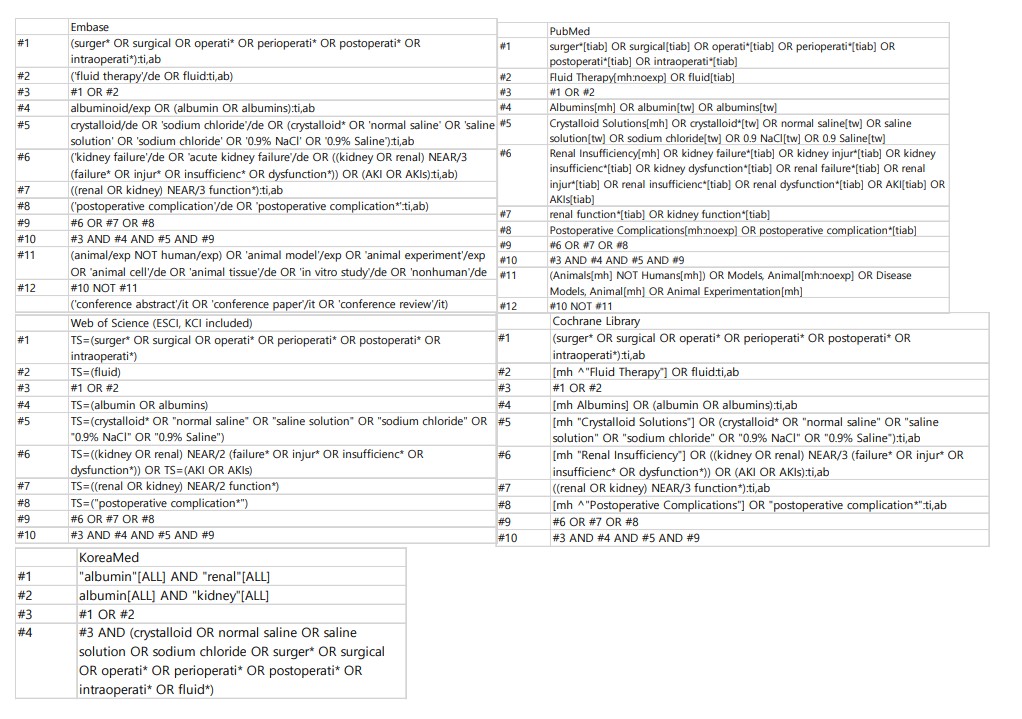

Supplement: Supplementary file 1 — Supplementary Figure 1. [file 41598_2024_62495_MOESM1_ESM.jpg]

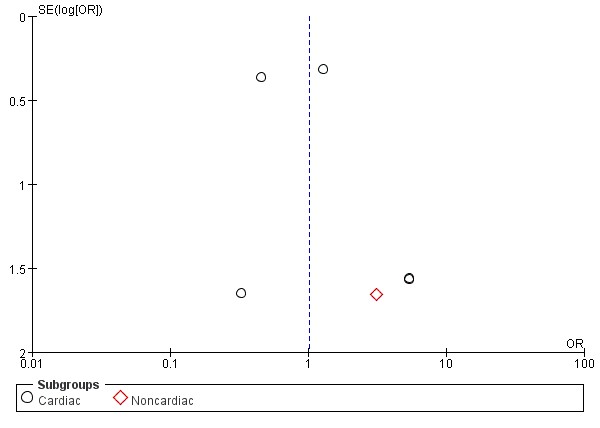

Supplement: Supplementary file 2 — Supplementary Figure 2. [file 41598_2024_62495_MOESM2_ESM.jpg]
