# Supplementary material for: The effect of human albumin administration on postoperative renal function following major surgery: a systematic review and meta-analysis
Source: Sci Rep. 2024 Jul 18;14:16599. doi: 10.1038/s41598-024-62495-0 (PMC11258253; doi:10.1038/s41598-024-62495-0)
Supplement: Supplementary file 3 — Supplementary Table 1. [file 41598_2024_62495_MOESM3_ESM.docx]

Supplemental Table 1. Sensitivity analysis

| Excluded study | Synthetic colloid | Random effect, Odds ratio | P value |
| --- | --- | --- | --- |
| Lee 2016 | used | 1.40 [0.79 to 2.51] | 0.25 |
| Abdallah 2014, Bisgaard 2020, Lee 2016, Skhirtladze 2013 | Used and not stated | 1.30 [0.71 to 2.37] | 0.40 |
